# Role of Cytokinin, Strigolactone, and Auxin Export on Outgrowth of Axillary Buds in Apple

**DOI:** 10.3389/fpls.2019.00616

**Published:** 2019-05-15

**Authors:** Ming Tan, Guofang Li, Xilong Chen, Libo Xing, Juanjuan Ma, Dong Zhang, HongJuan Ge, Mingyu Han, Guangli Sha, Na An

**Affiliations:** ^1^College of Horticulture, Northwest A&F University, Yangling, China; ^2^Institute of Agricultural Science, Qingdao, China; ^3^College of Life Science, Northwest A&F University, Yangling, China

**Keywords:** apple, axillary bud, cytokinin, auxin transport, strigolactone

## Abstract

Shoot branching is regulated by phytohormones, including cytokinin (CK), strigolactone (SL), and auxin in axillary buds. The correlative importance of these phytohormones in the outgrowth of apple axillary buds remains unclear. In this study, the outgrowth dynamics of axillary buds of a more-branching mutant (MB) and its wild-type (WT) of *Malus spectabilis* were assessed using exogenous chemical treatments, transcriptome analysis, paraffin section, and reverse transcription-quantitative PCR analysis (RT-qPCR). High contents of CK and abscisic acid coincided in MB axillary buds. Exogenous CK promoted axillary bud outgrowth in the WT but not in MB, whereas exogenous gibberellic had no significant effect on bud outgrowth in the WT. Functional analysis of transcriptome data and RT-qPCR analysis of gene transcripts revealed that MB branching were associated with CK signaling, auxin transport, and SL signaling. Transcription of the SL-related genes *MsMAX1, MsD14*, and *MsMAX2* in the axillary buds of MB was generally upregulated during bud outgrowth, whereas *MsBRC1*/*2* were generally downregulated both in WT and MB. Exogenous SL inhibited outgrowth of axillary buds in the WT and the apple varieties T337, M26, and Nagafu 2, whereas axillary buds of the MB were insensitive to SL treatment. Treatment with *N*-1-naphthylphalamic acid (NPA; an auxin transport inhibitor) inhibited bud outgrowth in plants of the WT and MB. The transcript abundance of *MsPIN1* was generally decreased in response to NPA and SL treatments, and increased in CK and decapitation treatments, whereas no consistent pattern was observed for *MsD14* and *MsMAX2*. Collectively, the present results suggest that in apple auxin transport from the axillary bud to the stem may be essential for the outgrowth of axillary buds, and at least, is involved in the process of bud outgrowth.

## Introduction

Shoot branching is important for tree structure, blossoming, and fruit yield management in apple. Shoot branching is a major determinant of aboveground plant architecture and occurs through the growth of axillary shoot meristems referred to as axillary buds. In both dicots and monocots, plant architecture is generally dependent on branching characteristics that result from interactions between genetic factors and environmental conditions. Shoot branching occurs throughout the growing period of a plant, with patterns of branching generally classified as monopodial (a new axillary meristem is formed at each node of the shoot axis), sympodial (a new apical meristem attached to the rudimentary terminal bud), or dichotomous (the terminal bud divides into two shoot apical meristems, or two new apical meristems at each node attached to the rudimentary terminal bud), and tillering (a new meristem forms attached to the basal portion of the stem in *Poaceae*) (McSteen and Leyser, 2005; Barthélémy and Caraglio, 2007). In apple trees, monopodial branching occurs during the vegetative stage, and sympodial branching during reproductive stages of the life cycle (Costes et al., 2014).

The outgrowth of axillary buds can be divided into four stages: initiation and formation of axillary buds, correlative inhibition (apical dominance), induction (bud activation), and sustained growth leading to axillary branching. Each of these stages are regulated by a different process, and are affected by different hormones (Waldie et al., 2010). The different stages in axillary bud development are generally existed, but there are no clear separated points among stages (Shimizu-Sato and Mori, 2001; Barbier et al., 2015). The axillary buds of apple, located in the leaf axil, are generally dormant due to the correlative inhibition exerted by apical buds (Cline, 1997; Olesen and Muldoon, 2009). Outgrowth of axillary buds (branching) in apple typically occurs during spring budburst following winter dormancy (Cook et al., 2001).

Combined genetic, physiological, and molecular evidence indicates that branching is regulated by a number of signals, including sucrose, light, nutrients, and phytohormones (Gonzalez-Grandio et al., 2013; Rameau et al., 2015; Wang et al., 2019). Exogenous auxin or endogenous auxin produced in stem tissues indirectly inhibit branching, and involving the expression of SL biosynthesis genes and repression of CK biosynthesis in stem tissues (Chatfield et al., 2000; Brewer et al., 2009; Young et al., 2014). CK application to axillary buds or shoots stimulates the outgrowth of axillary buds (Cook et al., 2001; Shimizu-Sato et al., 2009; Waldie and Leyser, 2018). Our recent study showed that exogenous CK and decapitation could induce the activation and outgrowth of axillary buds in apple, and suppression of CK synthesis resulted little axillary branches after decapitation (Li et al., 2018). Sucrose triggers initiation of axillary bud outgrowth in *Rosa, Arabidopsis*, and *Pisum*, whereas the response is not dependent on CK (Barbier et al., 2015). CK application to axillary buds or increasing CK biosynthesis, however, are insufficient to induce branching (Barbier et al., 2015). Spring budburst in apple is specifically triggered by CKs from the shoot (Cook et al., 2001). SL, a secondary messenger of auxin that directly regulates axillary bud outgrowth, acts in a species-conserved manner to inhibit lateral bud outgrowth and a feedback regulatory mechanism exists between the SL biosynthesis and SL response pathways (Hayward et al., 2009). SL synthesis occurs throughout the apple plant (Yue et al., 2015). Moreover, *MAX3* and *MAX4*, which are involved in SL synthesis in apple, show conserved functions within *Arabidopsis*, and complement the corresponding mutants (Arite et al., 2007; Foster et al., 2017).

In addition to the known signals, a recent review systematically analyzed the central role of TCP transcription factors in inhibition of shoot branching (Wang et al., 2019). Expression of class II TCP transcription factors, *BRC1*/*TB1*/*FC1*, is influenced by known signals, and is considered to be an integrator of diverse hormonal signaling networks (Wang et al., 2019). Previous studies have focused on clarifying the molecular mechanism between *BRC1*/*TB1*/*FC1* and the known signals, and observed that promotion and inhibition of branching are not always linked with the downregulation and upregulation of *BRC1*/*TB1*/*FC1*, respectively (Minakuchi et al., 2010; Muller and Leyser, 2011; Braun et al., 2012; Wang et al., 2019). Therefore, the mechanism by which *BRC1* expression is mediated by known signals remains incompletely understood.

The physiological and molecular mechanisms of SL and polar auxin transport (PAT) underlying axillary bud outgrowth have been extensively studied. Two models attempt to explain SL and PAT on the basis of hormone contents and expression of hormone-related genes. One model is known as the second messenger-based model, in which bud outgrowth can be directly repressed in response to increased contents of SL and exogenous application of GR24, a synthetic SL (Brewer et al., 2009). The second model is based on auxin canalization and PIN-FORMED 1 (PIN1) localization in axillary buds (Sachs, 1981). This model proposes that the formation of vascular connections from the axillary buds to the stem is crucial for bud outgrowth, which is dependent on decreased PAT or auxin content in the stem relative to that of the axillary buds. The occurrence of a greater number of branches in the *decreased apical dominance1* (*dad1*) of petunia, which shows reduced PAT compared with that of wild-type plants, is contradictory to the second model (Napoli, 1996). Contradictory relative PAT intensities have also been observed between wild-type plants and different more-branching mutants (Prusinkiewicz et al., 2009). Several experiments have demonstrated that PAT in the stem is not correlated with shoot branching (Brewer et al., 2015).

Interestingly, SL has an inhibitory effect on the polar subcellular localization of PIN1 in axillary buds that is differs from the largely SL-independent regulation of auxin transport and CK biosynthesis in stems (Shinohara et al., 2013; Young et al., 2014). This effect is rapidly detected after GR24 treatment and is dependent on clathrin-mediated membrane trafficking, which is also dependent on the function of MAX2 (Shinohara et al., 2013). *MAX2* encodes an F-box protein plays crucial roles in a variety of important biological processes, such as photomorphogenic development, leaf development, plant resistance, and shoot branching (Stirnberg et al., 2002, 2007; Shen et al., 2012; Piisila et al., 2015). The MB mutant of apple showed phenotypic traits involving weak viability and increased number of branches (Li et al., 2016), which suggests that MB phenotypes may be associated with the function and expression of *MsMAX2*. However, the function of apple *MAX2* on SL-mediated shoot branching is reserved to be identified.

To clarify the importance of SL, auxin transport, and CK in shoot branching in apple, hormone treatments, paraffin section, transcriptome analysis (RNA-seq), and reverse transcription-quantitative PCR (RT-qPCR) were conducted in this study. We examined transcriptomic differences in axillary buds before outgrowth in the WT, and the MB mutant of ‘Bly114’ apple, which exhibits both increased number and multi-level branches during the growing season (Li et al., 2016). Changes in branching phenotypes and gene expression in the WT and MB were comprehensively analyzed in response to decapitation, and application of GR24 and NPA (Dai et al., 2006; Brewer et al., 2015). Based on the present results, we hypothesize that auxin transport from the axillary bud to the stem and SL signaling are important modulators of hormone signals and gene expression during apple branching.

## Materials and Methods

### Plant Material, Growth Conditions, Treatments, and Sampling

A more-branching mutant (MB) and its wild-type (WT) of ‘Bly114’ apple (*Malus spectabilis*) were obtained from apple germplasm nursery of the Institute of Agricultural Science of Qingdao (36°24′N, 120°58′E), China. The mutant was originally selected from a branch exhibiting natural variation on a WT plant. The scions of WT, MB, and three additional apple varieties, namely T337, M26, and ‘Fuji’ Nagafu 2 (*Malus domestica*), were grafted onto 2-year-old shoots of wild apple (*Malus robusta*) every year from 2014 to 2016. It must be stated that the growth and phenotypes of each genotype-maintained consistency both in Yangling and Qingdao. The grafted plants were cultivated in a field at Yangling (34°52′N, 108°70′E), Shaanxi, China, with periodic water, fertilizer, and pest management treatments. For details, nitrogenous fertilizer was applied at the end of February, and plant nutrient solution containing large and trace elements was applied twice every 15 days in May; imidacloprid was applied to controlled aphid every 10 to 15 days from March to May, and pyrethroids were applied to control leaf pests from May to July.

To explore the effects of SL and PAT on axillary bud outgrowth, exogenous chemicals were applied directly to the axillary buds at 60–70 DAB, and the intact trees were approximately 1.2 m height. Young stems of experimental trees of WT MB, T337, M26, and Nagafu 2 were decapitated (i.e., the young and unlignified stem was removed). A drop (approximately 20–25 μl) of GR24 (Chiralix, Nijmegen, Netherlands) applied to axillary buds of the decapitated WT, T337, M26, and Nagafu 2, ranged in concentration from 0 to 1 mM (containing 0.1% Tween-20 and 0.3% DMSO). GR24 was applied at 12 h intervals for 7 days to screen the application concentration. Subsequently, a drop 0 or 20 μM GR24 was applied to axillary buds of MB and WT for 7 days. In addition, a drop of solvent (1% PEG6000, 0.1% Tween-20, and 0.5% DMSO) with 0 or 1 mM NPA was applied to axillary buds following decapitation (Barbier et al., 2015; Brewer et al., 2015) at daily intervals for 7 days.

To determine the effect of exogenous CK application, 5 mM solutions of 6-benzylaminopurine (6-BA) and gibberellic acid (GA_3_) (Sigma-Aldrich, St Louis, MO, United States) were applied to axillary buds of intact (no decapitation) WT for once at the same time as GR24 and NPA treatments. The 6-BA and GA_3_ were prepared by first dissolving GA_3_ in ethanol and 6-BA in 0.5 M NaOH (Ni et al., 2015).

Samples of the axillary buds and root apexes were harvested from 10 to 15 plants from each biological replicate from March to June, 2016 for RNA extraction. In addition, the samples of axillary buds for measurement of phytohormone contents and RNA sequencing (RNA-seq) analysis were collected at 60 DAB.

### Measurement of Morphological and Anatomical Phenotypes

The plant height, stem diameter, node number, branch number, branch angle, and bud length were recorded for WT and MB trees.

After decapitation terminal bud by pinching, axillary buds on the young stems were collected for paraffin embedding and thin sectioning using a previously described protocol (Naija et al., 2008).

### Measurement of Hormone Levels

High-performance liquid chromatography was used to quantify the contents of indole acetic acid (IAA), zeatin riboside (ZR), abscisic acid (ABA) and GA_3_, in 0.2 g of freshly collected axillary buds at 60 DAB accordance with previously described methods (Xing et al., 2015). Samples consisted of three biological replicates each comprising three technical replicates.

### Total RNA Isolation

Total RNA was isolated from each sample using a previously reported method modified by the addition of a purification step, and cDNA was synthesized as previously described (Xia et al., 2012). The integrity of RNA was evaluated by agarose gel electrophoresis and RNA concentrations were determined using a NanoDrop^TM^ 2000c spectro-photometer (NanoDrop Technologies, Wilmington, DE, United States).

### RNA-Seq and Mapping

Total RNA extracted from axillary buds at 60 DAB was subjected to RNA-seq. The RNA-seq has been performed in triplicate. Each replicate consisted of 30–40 buds from 15 plants (two to three buds per plant). After treatment with DNase I to eliminate contaminating DNA, mRNA was purified from total RNA extracts using magnetic oligo (dT) beads. The mRNA was mixed with fragmentation buffer and cleaved into short fragments for use as templates for cDNA synthesis. Short fragments were purified, resolved with buffer for end reparation and single adenine nucleotide addition and connected with adapters. After agarose gel electrophoresis, suitable fragments were selected as templates for PCR amplification. An Agilent 2100 Bioanalyzer and an ABI StepOnePlus^TM^ Real-Time PCR system were used for quantification and quality assessment of sample libraries. The constructed libraries were sequenced using an Illumina HiSeq^TM^ 2000 system (BGI, Shenzhen, China).

Primary sequencing data (raw reads) produced on the Illumina system were filtered into clean reads that were aligned to the apple reference genome (*Malus* × *domestica*, GDDH13 Version 1.1 2017) (Daccord et al., 2017). The mapping methods (mapping ratio and unique mapping ratio of clean reads) were identical to those used in a previous study (Li et al., 2018).

### Identification, Functional Annotation, and Pathway Enrichment Analysis of Differentially Expressed Genes

Unigene expression was calculated using the fragments per kilobase of transcript per million (FPKM) mapped Reads method (Mortazavi et al., 2008). The total clean tag number of all samples was digitally normalized after filtering and completed using EdgeR (Robinson et al., 2010; McCarthy et al., 2012). DESeq2 was used to estimate false discovery rates (FDRs) for differential expression by describing a number of applications possible with shrunken fold changes and their estimates of standard error (LoveI et al., 2014). DEGs between MB and WT were identified according to the following criteria: FDR < 0.001 and | log_2_ (fold change)|≥ 1 (Benjamini and Yekutieli, 2001).

The DEGs were annotated using the Clusters of Orthologous Groups of proteins (COG) database of protein clusters from genomes of unicellular and eukaryotic organisms (Tatusov et al., 2000). Based on the whole genome background of apple, Kyoto Encyclopedia of Genes and Genomes (KEGG) pathway enrichment analysis was performed using the KEGG database^1^ to identify significantly enriched metabolic or signal transduction pathways (Kanehisa et al., 2008). MapMan software was used to display expression profiles at the pathway level (Thimm et al., 2004).

### Quantitative Real-Time PCR

Specific primers for reverse transcription-quantitative PCR (RT-qPCR) were designed using Primer 6 software (Supplementary Table S1). To determine the relative expression level of the target genes, PCR amplifications were performed in a 20-μl volume using the SYBR^®^ Premix Ex Taq^TM^ II (Tli RNaseH Plus) Kit, with 10 μl of 2× SYBR Premix Ex Taq II (Takara, Beijing, China), and 1 μl of forward and reverse primers on a CFX Connect^TM^ Optics Module (Bio-Rad, Singapore). The thermal cycling protocol consisted of 95°C for 180 s, followed by 39 cycles of 95°C for 15 s, 58°C for 20 s, and 72°C for 20 s, followed by 39 cycles to construct a melting curve. The *ACTIN* (*MD04G1127400*) and *EF-α* (*MD04G1011300*) were used as internal references for normalization of gene expression in all samples. Each reaction was performed in three biological replicates. The expression of selected genes as determined by RT-qPCR was compared with the RNA-seq reads per million mapped reads data for these genes.

### Statistical Analysis

Statistical analysis, graphs, and figures were generated with Microsoft Excel 2007, Microsoft PowerPoint 2007, Origin Pro 7.5, and Adobe Photoshop CS5. Treatment effects were evaluated by one-way ANOVA and the significance of differences between the means of WT and MB was determined using Student–Newman–Keuls test and paired or unpaired samples *t*-tests as implemented in IBM SPSS Statistics Version 19 (Armonk, NY, United States).

## Results

### Phenotype of the Wild-Type and More-Branching Mutant Plants

**FIGURE 1:**
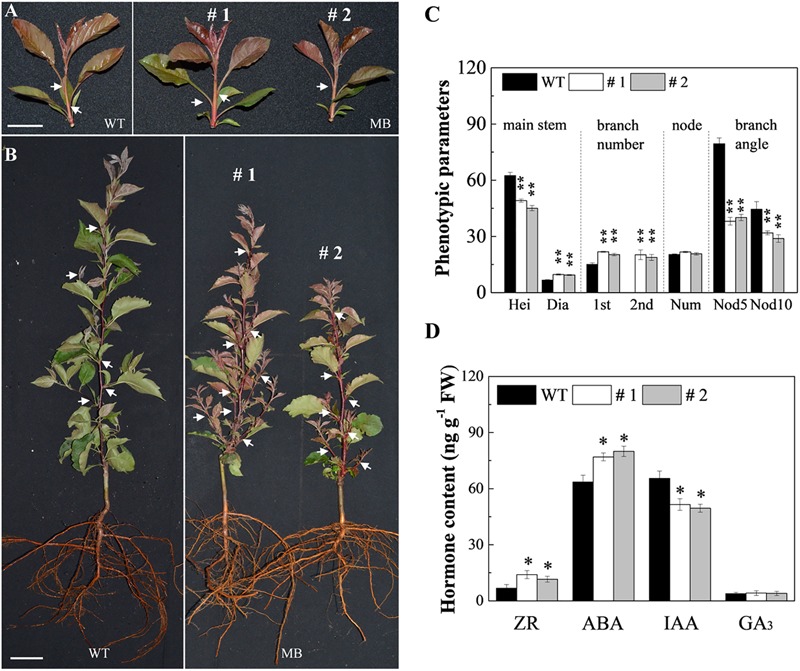
Branching phenotypes of the WT and MB. Branching phenotype of the primary shoot of the WT and MB at 25 days after bud break (DAB) **(A)** and 60 DAB **(B)** (Li et al., 2016). White arrows indicate the axillary buds or branches of partial nodes. **(C)** Stem height (Hei, cm), and stem diameter (Dia, mm), number of primary branches (1st) and secondary axillary branches (2nd), number of nodes (Num), and branch angle with stem (°) at nodes 5 (Nod5) and 10 (Nod10) at 60 DAB. **(D)** Zeatin riboside (ZR), abscisic acid (ABA), indole acetic acid (IAA), gibberellic acid (GA_3_) contents in the axillary buds of WT and MB at 60 DAB. #1 and #2 indicate different lines. Data represent the mean ± SE (**C**, *n* = 10 independent plants; **D**, *n* = 3). Significant differences between WT and MB plants are based on unpaired samples *t*-test (compared with WT, ^∗∗^*P* < 0.01, ^∗^*P* < 0.05). Scale bars = 2.5 cm **(A)**, 3.2 cm **(B)**.

To quantify the branching characteristics of WT and MB, the phenotype data was measured and analyzed. Branch number, plant height, bud length, stem diameter, leaf size, and branch angle between shoot and branch were initially similar in the WT and MB, but significant differences were observed at 60 DAB (Figure 1A,B and Supplementary Figure S1; Li et al., 2016). Compared with those of the WT, MB axillary buds displayed a significant increase in frequency of outgrowth and development of secondary branches, a reduced branch angle, and produced longer branches arising from the stem base (Figure 1C and Supplementary Figures S1B–E). The MB plants were shorter (Figure 1C) and produced smaller leaves (Supplementary Figure S1D) compared with those of the WT.

### Cytokinin Content and Role in Axillary Buds of WT and MB

**FIGURE 2:**
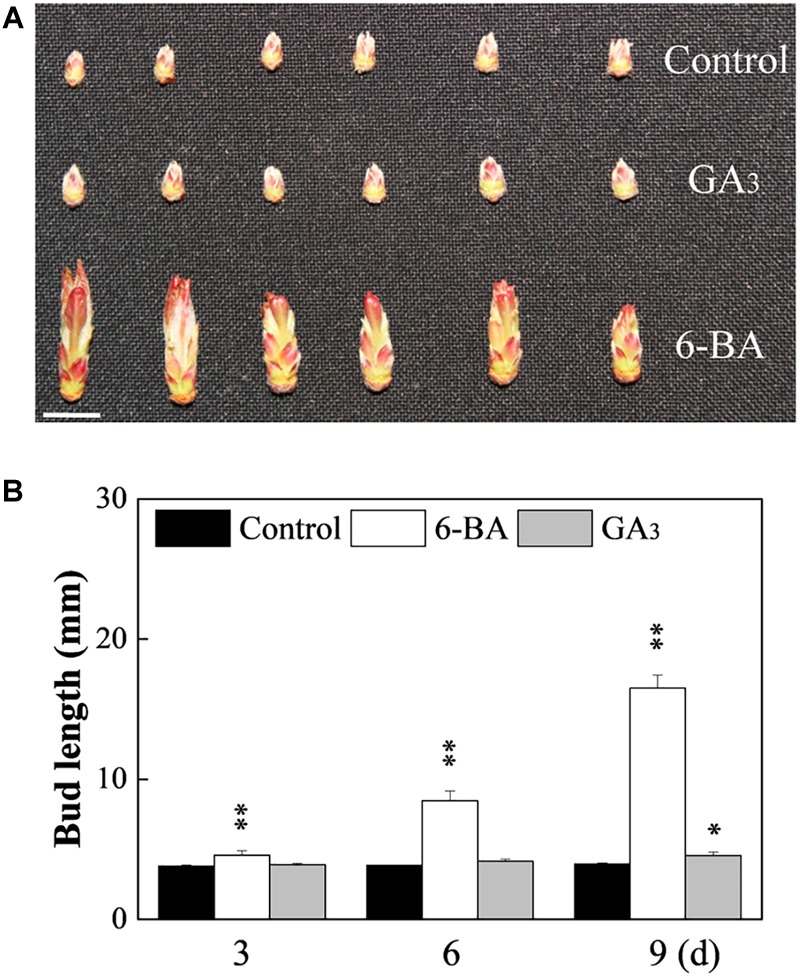
Effects of cytokinin and gibberellic acid on axillary bud outgrowth of WT. **(A)** Phenotype after treatment with cytokinin (6-BA) or gibberellic acid (GA_3_). Bud appearance at 9 days after treatment. **(B)** Bud length at 3, 6, and 9 days (d) after treatment. Data represent the mean ± SE (**A**, *n* = 3 replicates; **B**, *n* = 30 buds). Significant differences are based on a paired samples *t*-test (compared with control, ^∗∗^*P* < 0.01, ^∗^*P* < 0.05). Scale bars = 1.0 cm **(A)**.

To clarify the effect of CK on axillary buds of no outgrowth in the WT and MB, hormone contents and effect of exogenous CK were analyzed. The IAA content was significantly lower, whereas ZR and ABA contents were higher, in the axillary buds of the MB mutant compared with those of the WT at 60 DAB (Figure 1D). The contents of GA_3_ were similar in axillary buds of the WT and MB (Figure 1D). Outgrowth of axillary buds in the WT was significantly stimulated by application of 6-BA compared with the effect of GA_3_ (Figure 2). These results indicated that CK was positively associated with bud outgrowth in apple. The treatment of MB with 6-BA did not promote the number of branches in the same period, but increased the bud size to some extent (Supplementary Figure S1F).

### RNA-Seq and Functional Classification of Differentially Expressed Genes

**FIGURE 3:**
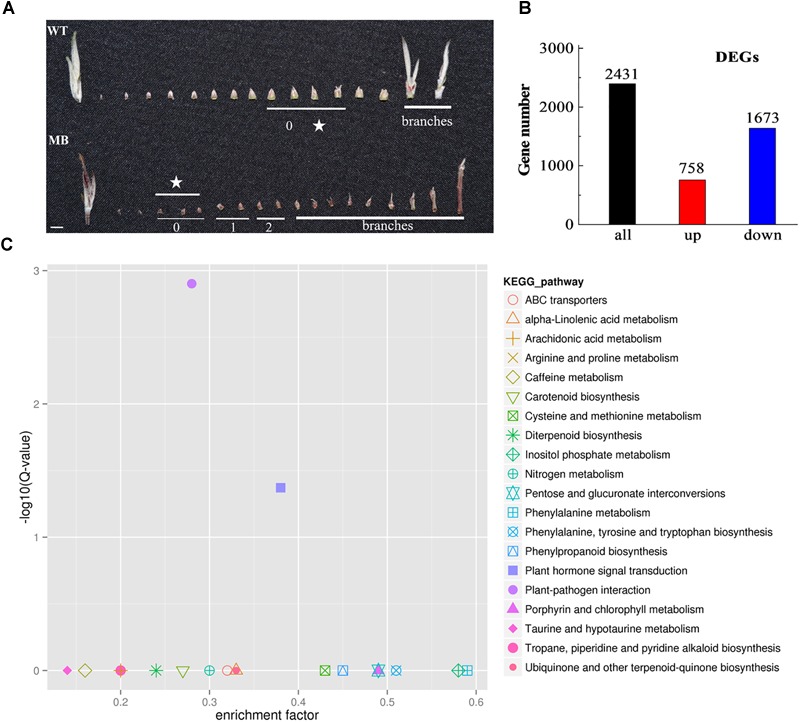
RNA-seq samples of axillary buds, and DEGs and KEGG analysis of the WT and MB. **(A)** Axillary bud phenotypes from the shoot apex to the branches in WT and MB at 60 DAB. The number under the bars indicates the relative stage of axillary bud development, ranging from 0 (dormant with no visible signs of growth) to 2 (visible signs of early outgrowth). The star indicates buds used for RNA-seq. Scale bar = 5.0 mm. **(B)** Number of DEGs that were significantly up- and down-regulated. **(C)** Enriched KEGG pathways of DEGs identified in the axillary buds of WT and MB. Results represent the comparisons of MB vs WT. Each symbol represents a KEGG pathway and the pathway name is listed in the right illustration. The abscissa represents the enrichment factor, which is calculated by comparing the gene ratio of one pathway to all genes with the ratio of DEGs annotated in one pathway to all DEGs. The ordinate represents the corrected multiple hypothesis testing, *Q*-value.

To characterize differential patterns of gene expression, axillary buds of no outgrowth selected from MB and WT plants at the branching stage (60 DAB; Figure 3A) were subjected to RNA-seq analysis. The mapping ratio and unique mapping ratio were 94.11–94.72% and 81.39–82.66% (Supplementary Table S2), which indicated a high credibility in base call accuracy.

The data indicated that 758 DEGs were up-regulated and 1673 DEGs were down-regulated in MB compared with the WT (Figure 3B). Analyses against the COG and KEGG databases were conducted to assess their potential biological relevance. The COG function analysis revealed that a majority of DEGs belonged to the transcription, replication, recombination and repair, and signal transduction mechanisms functional groups (Supplementary Figure S2). The KEGG pathways of plant–pathogen interaction and plant hormone signal transduction were significantly enriched among DEGs (Table 1 and Figure 3C).

**Table 1:** KEGG pathways of DEGs in the axillary buds of WT and MB.

Pathway	Pathway ID	*Q*-value
Plant–pathogen interaction	ko04626	8.575964e-33
RNA polymerase	ko03020	6.808301e-20
Pyrimidine metabolism	ko00240	2.352571e-14
Purine metabolism	ko00230	8.620354e-12
Sesquiterpenoid and triterpenoid biosynthesis	ko00909	9.095001e-05
Plant hormone signal transduction	ko04075	5.456657e-03

To validate the RNA-seq data, 26 genes associated with bud outgrowth were selected for RT-qPCR analysis. The linear correlation between RNA-seq and RT-qPCR was 81.25% (Supplementary Figure S3).

### Transcripts of Hormone-Related Genes During Axillary-Bud Outgrowth

**FIGURE 4:**
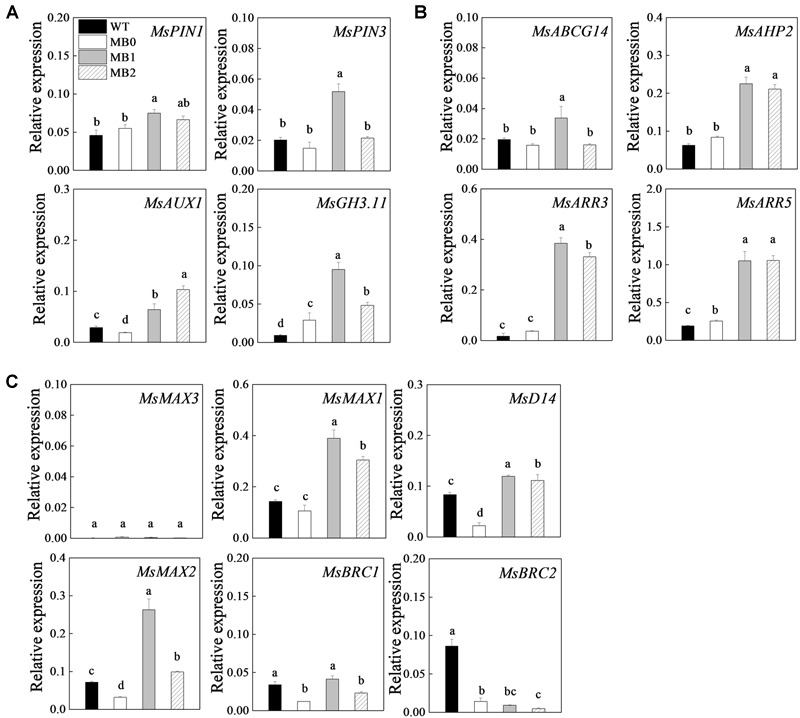
Expression of hormone-related genes during axillary bud outgrowth in MB. Genes associated with auxin transport and response **(A)**, CK transport and signaling **(B)**, and SL synthesis and signaling **(C)**. Data represent the mean ± SE (*n* = 3 replicates). Lowercase letters indicate significant differences between the WT and the different stages of axillary bud outgrowth in MB plants as determined by a Student–Newman–Keuls test (*P* < 0.5). WT, MB0, MB1 and MB2 indicate the stages of bud development indicated in Figure 3A.

The transcripts of hormone-related genes, selected on the basis of their expression profiles in the RNA-seq data and previous studies (Muller and Leyser, 2011; Janssen et al., 2014; Young et al., 2014), were analyzed in axillary buds of the MB mutant before and during outgrowth (Figure 4 and Supplementary Table S3). The auxin transport genes *MsPINs* and *AUXIN-RESIST-ANT1* (*MsAUX1*) (Young et al., 2014; Bennett et al., 2016), and the auxin response gene *GRETCHENHAGEN 3.11* (*MsGH3.11*) (Takase et al., 2003), were up-regulated at stage “1” compared the transcript levels of the WT and stage “0” (Figure 4A). *ATP-BINDING CASSETTE G14* (*MsABCG14*), a CK transport gene (Zhang et al., 2014), was up-regulated from stages “0” to “1” (Figure 4B). Interestingly, the CK signaling genes, *HISTIDINE-CONTAINING PHOSPHOTRANSMITTER 2* (*MbAHP2*), type-A *RESPONSE REGULATOR 3* (*MbARR3*) and *MbARR5* (Azizi et al., 2015), were significantly up-regulated in MB after stage “0” compared with the transcript levels in the WT (Figure 4B). These results indicated that expression levels of the selected genes in axillary buds of the MB were significantly activated after stage “0,” as outgrowth of the buds progressed.

The SL signaling genes *DWARF 14* (*MsD14*) and *MORE AXILLARY BRANCHES 2* (*MsMAX2*) were increased in axillary buds of the MB at stage “1” and “2,” and in root apex (Janssen et al., 2014) (Figure 4C and Supplementary Figure S4). In addition, the transcript frequency for *MsMAX2* decreased at stage “2” to 38.82% compared with that at stage “1.” The SL synthesis gene *MsMAX1* was also up-regulated both in axillary buds and roots. *MrMAX3*, an additional crucial SL synthesis gene, was down-regulated in roots and transcripts were not detected in axillary buds. Compared with WT plants, *MsBRC1* and *MsBRC2*, members of the class II TCP family associated with SL signaling were significantly down-regulated in MB except for *MsBRC1* at stage “1” (Figure 4C). Given that SLs inhibit shoot branching, these results suggested that SL signaling genes were up-regulated in roots and axillary buds at stage “1” in the MB mutants.

### Transcripts of Genes Associated With Meristem Development, Cell Proliferation, and Cell Growth During Axillary-Bud Outgrowth

**FIGURE 5:**
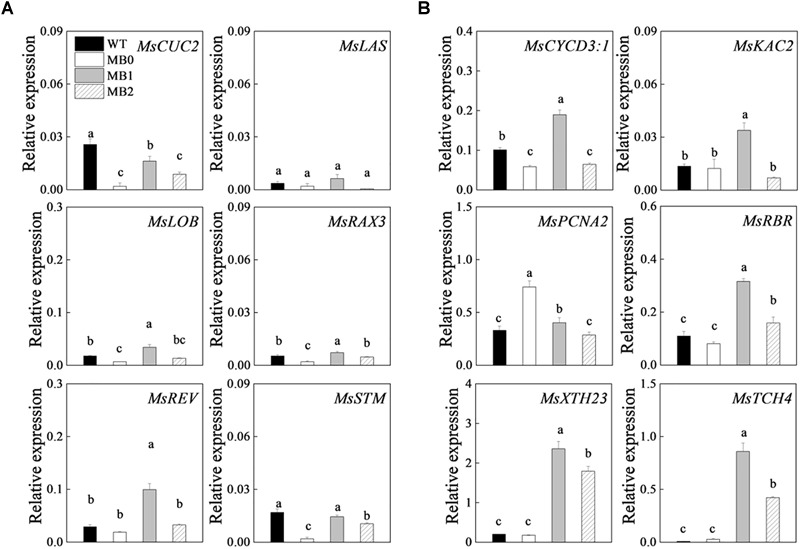
Expression of axillary meristem-related **(A)** and cell proliferation- and growth-related **(B)** genes during axillary bud outgrowth. Data represent the mean ± SE (*n* = 3 replicates). Lowercase letters above columns indicate significant differences between the WT and the different stages of axillary bud outgrowth in MB plants as determined by the Student–Newman–Keuls test (*P* < 0.5). WT, MB0, MB1, and MB2 refer to the stages of bud development indicated in Figure 3A.

Changes in the transcript levels of axillary meristem-related and cell proliferation- and growth-related genes were examined to characterize the developmental stages of axillary bud outgrowth in the MB mutant. The expression of meristem development-related genes in the axillary meristem was relatively low (Figure 5A). The expression of cell proliferation genes, such as *CYCLIN D3;1* (*MsCYCD3;1*) and *KINESIN LIKE PROTEIN FOR ACTIN BASED CHLOROPLAST MOVEMENT 2* (*MsKAC2*) were up-regulated at stage “1” in MB, whereas the cell proliferation gene *PROLIFERATING CELL NUCLEAR ANTIGEN 2* (*MsPCNA2*) was down-regulated after stage “0” (Cubas et al., 1999; Suetsugu et al., 2010; Muller and Leyser, 2011) (Figure 5B). Cell growth-related genes, such as *TOUCH 4* (*MsTCH4*) and *XYLOGLUCAN ENDOTRANSGLUCOSYLASE/HYDROLASE 23* (*MsXTH23*) were up-regulated after stage “0” (Sasidharan et al., 2010) (Figure 5B). The transcriptional changes in cell division- and growth-related genes indicated that cell proliferation and growth preceded visible morphological changes (Figure 5B).

**FIGURE 6:**
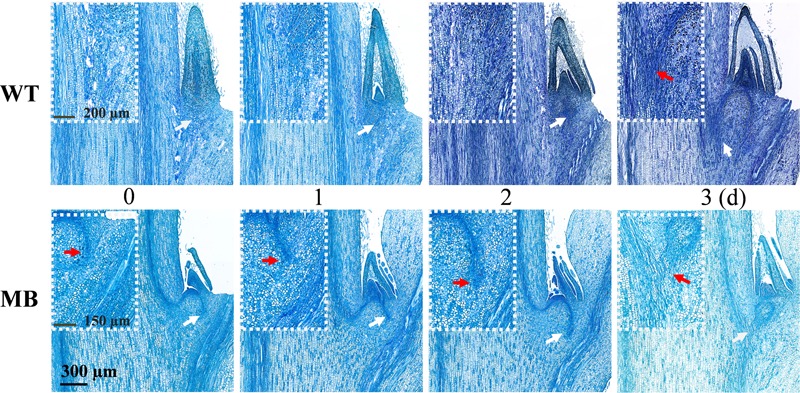
Development of vasculature from the axillary bud to the stem of outgrowth axillary buds of WT and MB. White arrows indicate the central position of white boxes, and red arrows indicate the visible vascular bundle from the bud. Numbers indicate days (d) following decapitation treatment.

To visualize the outgrowth process, longitudinal sections of axillary buds of the WT and MB were observed following pinching of the terminal bud (Figure 6). Internal structure of growth center in axillary buds was clearly changed, whereas the axillary bud size showed no visible change within 2 days in WT and MB. Vasculature from the axillary bud to the stem had clearly formed at day 1 in the MB and day 3 in the WT, which would mediate auxin flow from the axillary bud to the stem (Sauer et al., 2006; Prusinkiewicz et al., 2009; Bennett et al., 2014).

Collectively, the results showed that changes in gene expression were detected prior to visible signs of growth and changes in expression of cell growth-related genes appeared to be stronger than that of cell proliferation-related genes. The transcriptional expression of meristem development- and cell replication-related genes, however, was not synchronized; which may be indicative of separated stages in the process of axillary bud outgrowth (Shimizu-Sato and Mori, 2001; Barbier et al., 2015).

### Impact of GR24 and NPA on Axillary Bud Outgrowth and Gene Expression

**FIGURE 7:**
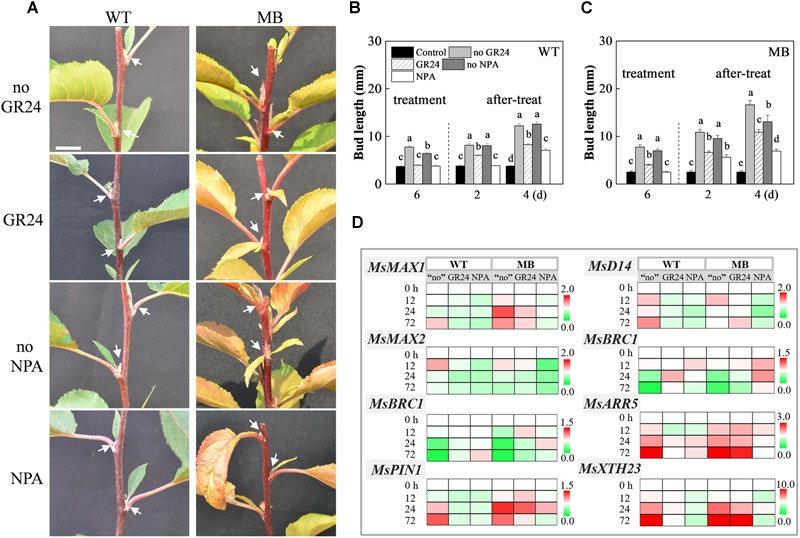
Branching phenotypes and gene expression in response to GR24 and NPA treatments of WT and MB. **(A)** Outgrowth of axillary buds in decapitated WT and MB in response to the exogenous application of the synthetic SL analog GR24, and the auxin polar transport inhibitor NPA. Arrows indicate the axillary buds or branches of nodes. Overall bud length in decapitated WT **(B)** and MB **(C)** in response to GR24 and NPA. “Control” represents the axillary buds with no outgrowth in intact plants **(B,C)**. “Treatment” is the period of sustained application of GR24 and NPA to the axillary buds for 7 days, and “after-treat” is the period after cessation of treatment **(B,C)**. Horizontal axis indicates the days (d) following treatments **(B,C)**. **(D)** Expression levels of *MsMAX1, MsD14, MsMAX2, MsBRC1, MsBRC2, MsARR5, MsPIN1*, and *MsXTH23* after treatments. All transcripts were normalized to their respective corresponding abundance at 0 h. The “no” represents the decapitated control without NPA and GR24. Lower-case letters above columns indicate significant differences as determined by the Student–Newman–Keuls test (*P* < 0.5; **B,C**, *n* = 30 buds; **D**, *n* = 3 replicates). Scale bar = 10 mm **(A)**.

To examine the effects of SL and auxin transport from the axillary bud to the stem on bud outgrowth, solutions of exogenous SL (GR24) and an auxin transport inhibitor (NPA) were applied to axillary buds of decapitated WT and MB plants. As shown in Figure 7A, bud elongation in WT plants was suppressed with GR24 treatment. The same response was observed for axillary buds of the apple varieties Nagafu 2, T337, and M26 (Supplementary Figure S5). No obvious elongation of axillary buds of the WT and MB was observed in the present of NPA (Figure 7A,C). Compared with the no GR24 and no NPA treatments groups, axillary bud outgrowth of the MB was still response to GR24, but the bud length was significantly shorter after treatment for 6 days (Figure 7A,C). These results indicated that the MB mutant might be insensitive to exogenous SL as suggested by previous studies (Hamiaux et al., 2012; Jiang et al., 2013), and was able to respond to GR24, which suggests that SL performs presently unknown functions. Interestingly, the suppressive effects of GR24 and NPA on axillary bud outgrowth were lost a few days after treatment ceased (Figure 7B,C).

Compared with the decapitated control, transcripts of *MsMAX1, MsMAX2*, and *MsD14* in the WT were decreased in abundance in response to both GR24 and NPA treatments (Figure 7D). These three genes were also down-regulated in MB in response to NPA treatment, whereas no consistent change in response to GR24 was observed in MB (Figure 7D). Abundance of *MsMAX1, MsMAX2* and *MsD14* transcripts fluctuated in response to exogenous CK, compared with that of GA_3_ (Supplementary Figure S6). These results indicated that the expression patterns of *MsMAX1, MsMAX2*, and *MsD14* might be uncorrelated with the stage of axillary bud outgrowth in apple. The transcription of *MsBRC1* and *MsBRC2* in the WT and MB were downregulated in the control during bud outgrowth (Figure 7D). In addition, transcription of *MsBRC1*/2 was downregulated during bud outgrowth in the WT in response to CK treatment relative to those in response to GA_3_ and the control (Supplementary Figure S6). The relationship between bud outgrowth and expression level of *MsBRC1*/2 showed a negative correlation, which was also observed in our previous study (Tan et al., 2018).

To assess the relationship between axillary bud outgrowth and gene expression, the transcript abundances of *MsARR5, MsPIN1*, and *MsXTH23*, which are positive response to bud outgrowth during axillary bud outgrowth of the MB, was analyzed (Figure 7D and Supplementary Figure S6). These three genes were up-regulated in WT and MB in response to decapitation and CK treatment, compared with NPA and GA_3_ treatments. The transcript of *MsPIN1* was decreased in WT under NPA treatment, and that in MB was decreased at 12 and 72 h, and *MsXTH23* in the WT and MB was down-regulated in response to NPA treatment. These results indicated that inhibition of auxin transport from the axillary buds strongly inhibited outgrowth of axillary buds in apple.

## Discussion

The MB apple mutant used in this study provides a valuable resource for exploration of the molecular mechanism of branching regulation in apple. Using the method of forward genetics, however, it is difficult to dissect the specific characteristics in woody plants. Based on analysis of the transcriptome of specific cells or tissues, RNA-seq is a suitable method to identify the main biological processes or pathways associated with branching (Shi et al., 2006; Xing et al., 2015). The recent publication of a high-quality *de novo* assembly of the apple genome provides a powerful database for RNA-seq in apple (Daccord et al., 2017). Differences in the RNA-seq data of axillary buds between the MB and WT indicated that plant hormone signal transduction was significantly enriched, which provided the initial foundation for the present study. The present results also indicated that hormone signal transduction involving SL, CK, and auxin plays a crucial role in apple branching and clarified the relative importance of different hormone signaling pathways.

### Cytokinin Plays Divergent Roles in Regulation of Axillary Bud Outgrowth

A complex regulatory is associated with the outgrowth of a dormant bud and its transformation into an actively growing shoot (Rameau et al., 2015). Although GA_3_ treatment significantly increased the axillary bud size, it was insufficient to promote outgrowth compared with the 6-BA treatment (Figure 2). This result was consistent with the role of GAs as repressors of branching in *Arabidopsis*, pea, and rice (Lo et al., 2008; de Saint Germain et al., 2013; Ljung et al., 2015), and opposite to the responses in *Jatropha* and *Rosa* (Choubane et al., 2012; Ni et al., 2015). The effect of the CK treatment on axillary bud outgrowth of the WT and the changes in hormone contents that occur in axillary buds of the MB indicates that CK activates the outgrowth of axillary buds in apple. Similar to reports for *Arabidopsis* and pea, our previous study demonstrated that CK can activate axillary buds when applied directly to the axillary buds in apple (Brewer et al., 2009; Tan et al., 2018). However, suppression of CK synthesis and signaling does not inhibit the sucrose-promoted outgrowth of axillary buds in *Rosa hybrida* (Barbier et al., 2015). The molecular mechanism by which CK regulates branching may differ among apple, pea, and *Rosa hybrida* (Cook et al., 2001; Barbier et al., 2015). At the least, the present data indicate that simple activation of the cell cycle- and cell growth-related gene expression is insufficient to activate buds in apple, and that CK must, therefore, do more than simply up-regulate these processes in apple axillary buds (Muller and Leyser, 2011).

Cytokinin may activate buds by either modulating auxin transport or by locally up-regulating auxin biosynthesis in the axillary buds (Muller and Leyser, 2011; Waldie and Leyser, 2018). Preventing auxin accumulation in the meristem results in diminished meristem activity, the latter of which is dependent on CK signaling (Zhao et al., 2010). Auxin was shown to repress *A-type RESPONSE REGULATOR* (*ARR*) genes directly in the meristem (Zhao et al., 2010). The auxin response factor ARF5 (AUXIN RESPONSE FACTOR 5) binds to the promoter of *ARR15* and suppresses gene expression, thereby allowing CK signaling to occur (Zhao et al., 2010). In the current study, the low content of auxin in axillary buds of the MB might be due to its polar export from the axillary buds (Figure 1D). This feedback loop may be modulated by auxin-independent CK synthesis driven by the addition of auxin supplied by the newly activated branches or by nutrient availability (Young et al., 2014).

In the present study, high CK content and the expression of CK-related genes in the axillary buds of MB mutant were consistent with axillary bud outgrowth (Figure 1D, 4B). Changes in abundance of transcripts for auxin transport and response genes, however, were inconsistent with the IAA content, possibly because of auxin transport from activated buds to the main stem. Interestingly, analysis of type-A *arr* and *isopentenyltransferase (ipt)* multiple mutants demonstrates that defects in CK response do not affect auxin-mediated bud inhibition, and that increased abundance of *IPT* transcripts is not needed for bud release following decapitation (Muller et al., 2015). The finding that type-A ARRs are required for CK-mediated bud outgrowth may explain the increased expression of *MsARR3/5* during bud outgrowth observed in the current study (Figure 4B). Furthermore, sucrose promotes sustained growth in a CK-independent manner, even though it increases stem CK content (Barbier et al., 2015). As proposed in the canalization-based model for shoot branching, bud outgrowth requires canalization of auxin transport from the bud to the stem (Sachs, 1981).

### The Equilibrium Model of Strigolactone on Auxin Transport From Axillary Buds

As demonstrated in the present study, CK can directly promote shoot branching (Figure 2), whereas SL inhibit branching both *in vitro* and *in vivo* (Gomez-Roldan et al., 2008). Given that quantification of SL content in plant tissues is both difficult and expensive, the expression of SL-synthesis and SL-signaling genes, together with characterization of the phenotypic changes that occur after GR24 application, were combined in the current investigation. As expected, neither GR24 treatment nor grafting buds onto non-decapitated (normal) shoots could restore the WT phenotype in the MB mutant (Figure 1B, 7C), which indicated that the MB mutant is insensitive to SL (Dharmasiri et al., 2005; Lin et al., 2009). The genes detected by both RT-qPCR and RNA-seq that were identified in the MB mutant were part of the auxin and SL pathways. However, the MB phenotype, characterized by elevated expression of auxin genes could be restored by application of GR24 to SL-deficient mutants (Jiang et al., 2013). Previous studies have indicated that deficiencies in SL synthesis and SL signaling can attenuate shoot gravitropism of rice by inhibiting auxin biosynthesis (Sang et al., 2014). This effect may be responsible for the decrease in branch angle in the MB mutant (Figure 1C and Supplementary Figure S1E). On the other hand, the decelerated growth rate of MB axillary buds indicated that the MB mutant could respond to the treatment of exogenous SL. Therefore, the phenotypes of the MB mutant used in the present study may be associated with alteration in SL signaling, downstream of SL perception. The up-regulation of SL pathway genes, combined with the outgrowth of axillary buds in the MB mutant, may be a result of feedback regulation within the SL pathway (Hayward et al., 2009). In the SL synthesis pathway, *MAX3*, a gene codes carotenoid cleavage dioxygenase and related to branching, locates upstream of MAX1 (Hamiaux et al., 2012). Therefore, the absence of *MAX3* expression in the axillary buds of the MB mutant does not contradict this association because SL is mainly synthesized in roots (Figure 4C and Supplementary Figure S4). Given that the more-branching phenotype of the MB mutant, the down-regulation of *MrMAX3* in roots may be due to the feedback inhibition of *MrMAX1* (Figure 1 and Supplementary Figure S4).

Some reports have suggested that SL act by rapidly dampening PIN1 polarization on the plasma membrane (Shinohara et al., 2013). PIN1 is located on the plasma membrane in activated buds, whereas in inhibited buds PIN1 does not accumulate (Balla et al., 2011). This observation may indicate that inhibition of branching by SL acts independently of PAT in the stem (Brewer et al., 2015). Systemically, higher SL levels reduce the number of active axillary buds and the steady-state levels of PIN1 on the plasma membrane (Shinohara et al., 2013). These findings seem consistent with the present results whereby low concentrations of SL (less than 20 μM) were insufficient to inhibit bud outgrowth (Supplementary Figure S5). Combined with the effects of GR24 and NPA treatments on axillary bud outgrowth in the WT and MB (Figure 7), these results suggest that auxin export from the axillary buds to the stem is involved in bud outgrowth in apple.

### Anatomical Changes in Axillary Buds From Dormancy to Outgrowth

Cells attain a specific stage of development in buds before entering a dormant state (Devitt and Stafstrom, 1995; Shimizu and Mori, 1998). Changes in gene expression (Figure 5B), cell proliferation, and cell growth occur in the axillary buds before morphological changes are evident. Interestingly, increased cell proliferation and cell growth are insufficient to activate buds (Muller and Leyser, 2011; Li et al., 2018). As previously discussed, it appears that the release of an axillary bud from dormancy should result in the activation of cell proliferation and cell growth, and lead to both PIN1 polarization and up-regulation of auxin transport from buds.

It is worth mentioning that application of a high concentration of IAA to an axillary bud does not induce outgrowth in pea (Vieten et al., 2005; Brewer et al., 2009), which suggests that the canalization for bud outgrowth does not simply rely on relative concentrations of auxin between the bud and the stem. Decapitation may increase CK content, then promote auxin transport from the axillary bud to the stem, thus promoting vascular patterning and bud outgrowth (Bennett et al., 2014; Young et al., 2014). Thus, the combined relative strength of CK signaling, SL, and auxin transport in buds may determine the bud state. However, the molecular mechanism by which SL signaling regulates PIN1 polarization, and that of CK signaling during bud outgrowth remain to be characterized.

## Conclusion

**FIGURE 8:**
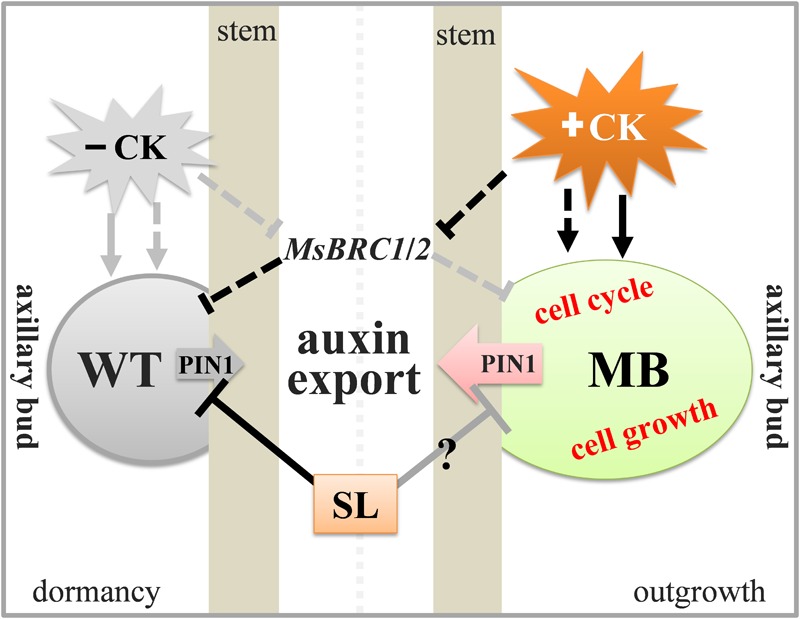
Schematic model of the effects of CK, SL, and auxin transport on axillary buds of the MB and WT apple. In the WT, SL and high expression of *MsBRC1*/*2* maintain dormancy of the bud. Expression of *MsBRC1*/*2* is suppressed by high CK, and the axillary bud is insensitive to SL (signaling) in MB. As a result, auxin transport from the axillary bud to the stem, the cell cycle, and cell growth are activated in MB, which induces bud outgrowth. Lines and gray boxes indicate non-effective roles; black lines and colored shading indicate effective regulations and processes.

The results of this study support the hypothesis that CK, SL, and auxin export from the axillary buds play crucial roles in apple branching. Importantly, auxin transport from the axillary bud to the stem may be essential during axillary bud outgrowth of in apple. The present results permit formulation of a schematic model for explanation of the diverse effects of different hormones on axillary buds in the WT and MB (Figure 8).

## Author Contributions

MT, GL, MH, JM, DZ, HG, GS, and NA participated in the experimental design. HG and GS provided the wild type and its more-branching mutant of ‘Bly114’ apple. MT, GL, XC, and LX performed material sampling, field measurements, measurement of laboratory data, and the analysis of RNA-seq data. MT, GL, MH, GS, and NA participated in the paper writing and manuscript amending.

## Conflict of Interest Statement

The authors declare that the research was conducted in the absence of any commercial or financial relationships that could be construed as a potential conflict of interest.

## Footnotes

1 http://www.genome.jp/kegg/

## Abbreviations

DAB: days after bud break
DEGs: differentially expressed genes
CK: cytokinin
MB: more-branching mutant
NPA: *N*-1-naphthylphthalamic acid
SL: strigolactone
WT: wild-type
