# Supplementary material for: Role of Cytokinin, Strigolactone, and Auxin Export on Outgrowth of Axillary Buds in Apple
Source: Front Plant Sci. 2019 May 15;10:616. doi: 10.3389/fpls.2019.00616 (PMC6530649; doi:10.3389/fpls.2019.00616)

Supplementary Table S1. Gene-specific primers used for RT-qPCR in *Malus***.**

| **Apple Genes Identification** | **Arabidopsis Homolog** | **Primers (5'-3')** |
| --- | --- | --- |
| *MD14G1236300* | *PIN1* | F: GCGGGATCCATTGTCTCCATCC  R: CTGAGACCGCCGCGAGAAAATA |
| *MD16G1027900* | *PIN3* | F: ACAATTCAACAGCAGCAGCA  R: CGGTCCCACCAAAAACATGG |
| *MD12G1162400* | *AUX1* | F: TCCTTAACCATTCCAACGCCTTCTC  R: GCCAATCACCTTCTCCCACACAAA |
| *MD07G1145900* | *GH3.11* | F: CGGAGCAATCTTCTGCCTTCAATCA  R: AGTGACCAGGATCTGTGGACAAGT |
| *MD08G1001300* | *ABCG14* | F: CCTCCAGCCGCATCTACCACAT  R: GCTCCTGTTCCGTGTTCGTATTCTC |
| *MD11G1293900* | *AHP2* | F: AGCTCTGCACTGAAGAACAAGC  R: TCGTAGGAATTGACCCGCCA |
| *MD16G1084400* | *ARR3* | F: TTGGAAGAAGGTGCGGAGGACTT  R: AACTGGACGGCGGTGATGGA |
| *MD14G1188400* | *ARR5* | F: AGGCGAGATTCTCAGGCGGAAT  R: TTAGCAGGCGTTCAATGACCTTCC |
| *MD07G1110600* | *MAX3* | F: TGGATAGCAACGGGAACTGTCAAAG  R: CAACGAACTGCGAGAACCTGAAGT |
| *MD15G1057600* | *MAX1* | F: GGCTTGATGGTATTGTGGC  R: ATGTGAATGCTGTGGTGGC |
| *MD08G1084800* | *D14* | F: ATTCAGCCGCACCCTGTTCAAC  R: CGTCCTTCGCCGTCTGGATTATC |
| *MD09G1236800* | *MAX2* | F: AGTGGGTTGGCGCTGGAAGT  R: AACGCAATGCCGTCGAGCTC |
| *MD06G1211100* | *BRC1* | F: AGCCCAGCAAAACCGTGGAGT  R: TGGAGCCTCATCGACGCCTG |
| *MD16G1049000* | *BRC2* | F: AGCACCTCCTCGTCTCACCCA  R: GCTCTTGCCTCCACCCTCGA |
| *MD11G1253500* | *CUC2* | F: CCGACGAGGAGCTTATCACCTACTA  R: TCGCCCATCTTTGCCTTGTCAG |
| *MD03G1045700* | *LAS* | F: CCGTCAACTGCGTCCTCTACCT  R: AGAGCCTCCACGAAGCGGTTAA |
| *MD06G1169000* | *LOB* | F: AGCGTGAGGATGCCGTGAACT  R: CTCTGGACCTGTCGTTGGAGGAA |
| *MD15G1302300* | *RAX3* | F: TGGTCACCTGAAGAAGATGCTACAC  R: AACCCGCCGTGCTTGATATTCG |
| *MD03G1118500* | *REV* | F: TGGTGACAACTCCTCAGCATTCTCT  R: CCAGGCTTCATCCCAGGCATCT |
| *MD05G1352500* | *STM* | F: TTGCCTCCGTCCAACAACCATC  R: GCTGCCGCCAACACCTTCATTA |
| *MD08G1092900* | *CYCD3;1* | F: GCGGTGGATTGGATGCTGAGAG  R: TGAAGTTGGAGGCTGGACAAGAAC |
| *MD15G1313500* | *KAC2* | F: ATGGATGGTTGGATGGCTGAACTG  R: TTGAGAACTGTAGACCCGCTTGGA |
| *MD07G1045800* | *RBR* | F: TCACTGCCATCAACAACCGCT  R: GGCCACCATCGAATCGCTGA |
| *MD03G1078300* | *PCNA2* | F: CACGGTCACAATCAGCTTGTCTTCT  R: GGCTTGGTTTCGTCTTCGTCATCT |
| *MD13G1237300* | *XTH23* | F: GCGGACTTGTGAAGACGGATTGG  R: GACAACGGGAAGTGCCTGAAGAC |
| *MD16G1267200* | *TCH4* | F: TCAACAACGGCGAGCTTCTTACC  R: TGTGACAGTGCCAGCGGAGTT |
| *MD16G1041700* | *IPT1* | F: GCCTCGACATCACCACCAACAAG  R: GGCACCTTCCTCCGATTCGTAATG |
| *MD03G1267900* | *IPT3* | F: TCGGTCCAAGTATGACTGTTGC  R: CCTCATCCACCATTCCGTTT |
| *MD04G1127400* | *Actin* | F: TGACCGAATGAGCAAGGAAATTACT  R: TACTCAGCTTTGGCAATCCACATC |
| *MD04G1011300* | *EF-1α* | F: ATTCAAGTATGCCTGGGTGC  R: CAGTCAGCCTGTGATGTTCC |

Supplementary Figure S1. Branch comparisons of WT and MB plants, and effect of 6-BA on axillary buds in the MB. (A) Comparison of a 2-year-old stem fragment before germination. (B**)** Branch number of one-year-old seedling. (C) Bud length (D), leaves (E) and branch angle at 75 DAG. (F) Bud phenotypes of MB response to 6-BA treatment below the shoot apex. Data are means ± SE (*n* = 15 plants). Significant differences (***P* < 0.01) are based on paired samples *t*-test. Scale bars = 8.0 mm (A), 4.0 cm (D), 4.0 cm (E) and 3.0 mm (F).


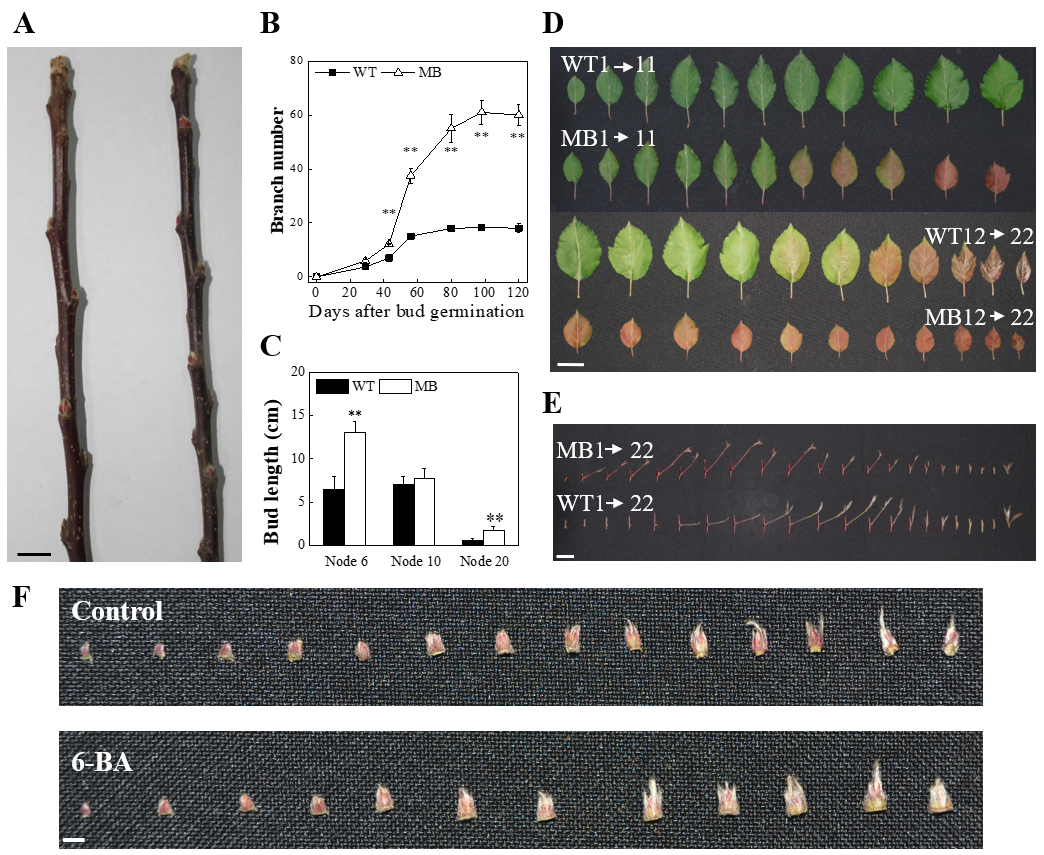


Supplementary Table S2. Summary of RNA-seq data from the axillary buds of WT and MB.

| **Sample Name** | **Clean reads** | **Mapped ration** | **Uniq mapped ration** |
| --- | --- | --- | --- |
| WT-a | 48285172 | 94.20% | 81.47% |
| WT-b | 46957348 | 94.72% | 82.66% |
| WT-c | 46897152 | 94.64% | 82.52% |
| MB-a | 48916890 | 94.12% | 81.39% |
| MB-b | 46954604 | 94.10% | 81.64% |
| MB-c | 46639714 | 94.11% | 81.48% |

Supplementary Figure S2. Cluster of Orthologous Groups of proteins (COG) representing DEGs identified in response to 6-BA application. Results represent the comparisons of MB vs. WT. The abscissa represents the detail content of COG and is indicated with an uppercase letter and corresponding color. The ordinate represents the frequency or more specifically the number of genes.


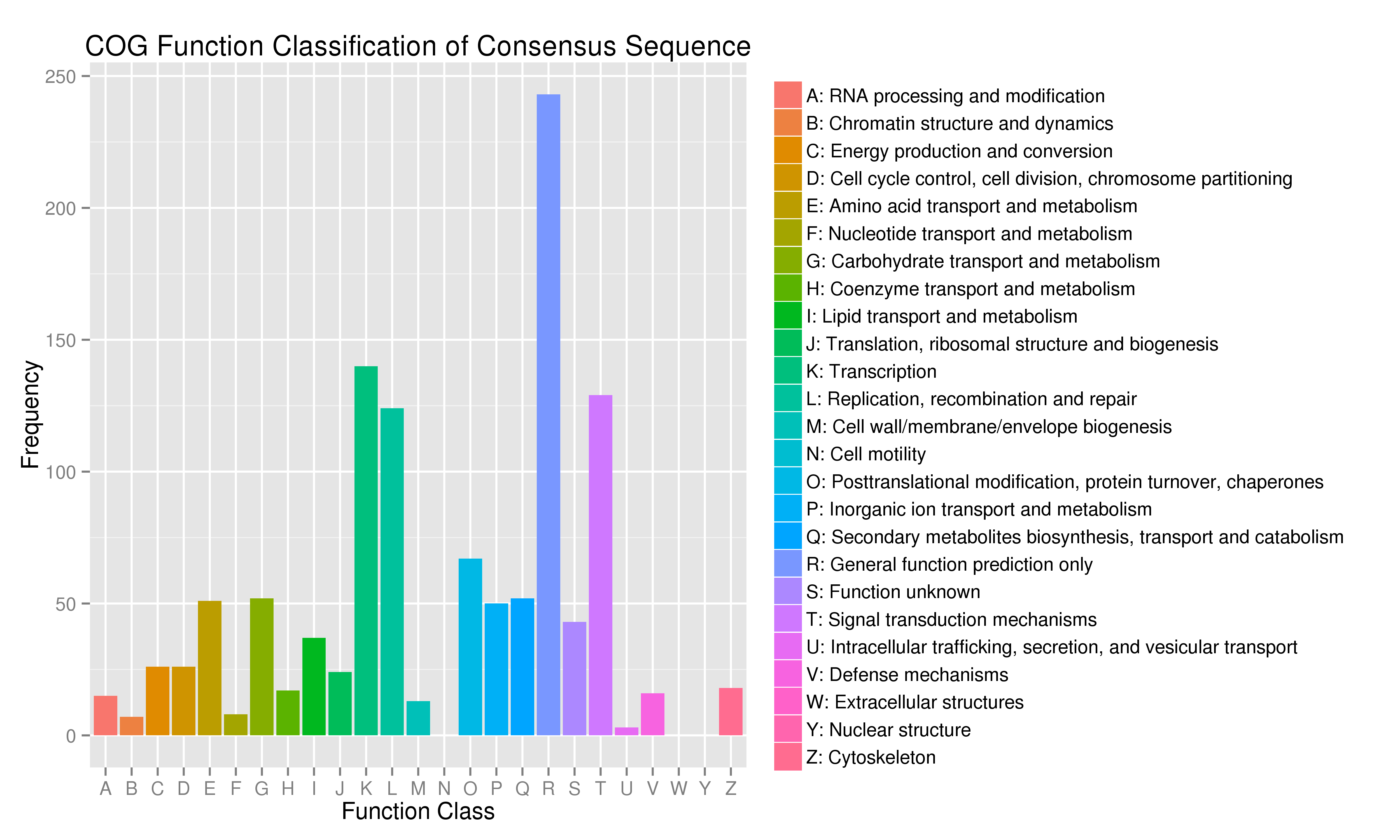


Supplementary Figure S3. Availability identification of the RNA-seq data. (A) Expression analysis of two reference genes, *ACTIN* and *EF-a*, in all samples. (B) Correlation identification of RT-qPCR and RNA-seq data using selected genes between MB and WT.


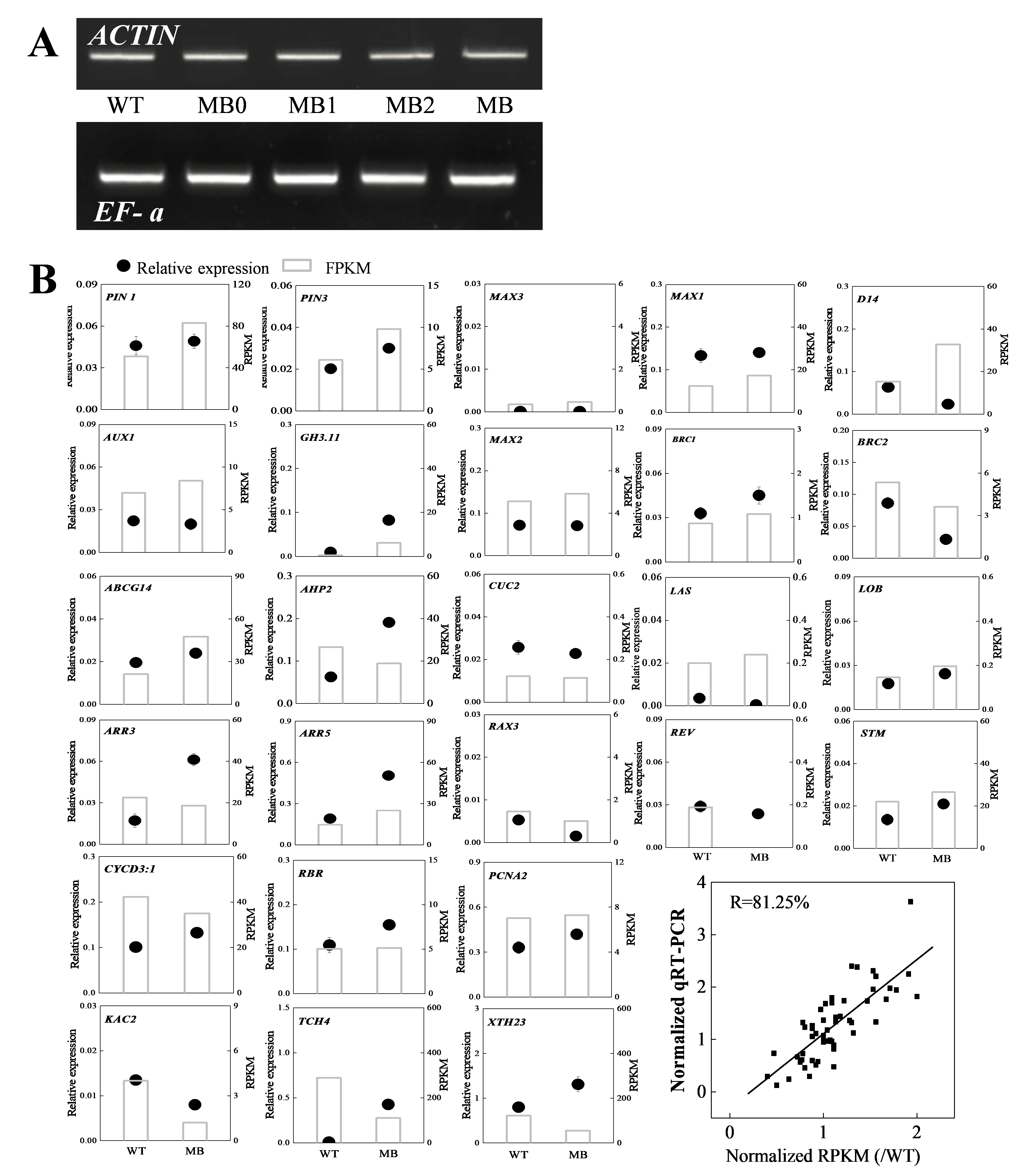


Supplementary Table S3. Annotation of the selected genes in *Malus* based on apple reference genome (https://iris.angers.inra.fr/gddh13/) and the *Arabidopsis* Information Resource (TAIR, <https://www.arabidopsis.org/index.jsp>).

| **Apple genes Identification** | **Arabidopsis Homolog** | **Names** | **Annotation** |
| --- | --- | --- | --- |
| **Hormone- auxin** | |  |  |
| MD14G1236300 | AT1G73590.1 | PIN1 | Auxin efflux carrier family protein |
| MD16G1027900 | AT1G70940.1 | PIN3 | Auxin efflux carrier family protein |
| MD12G1162400 | AT2G38120.1 | AUX1 | Transmembrane amino acid transporter family protein |
| MD07G1145900 | AT2G46370.4 | GH3.11 | Auxin-responsive GH3 family protein |
| **Hormone-** **cytokinin** | |  |  |
| MD16G1041700 | AT1G68460.1 | IPT1 | Isopentenyltransferase 1 |
| MD03G1267900 | AT3G63110.1 | IPT3 | Isopentenyltransferase 3 |
| MD08G1001300 | AT1G31770.1 | ABCG14 | ATP-binding cassette 14 |
| MD11G1293900 | AT3G29350.1 | AHP2 | histidine-containing phosphotransmitter 2 |
| MD16G1084400 | AT1G59940.1 | ARR3 | response regulator 3 |
| MD14G1188400 | AT3G48100.1 | ARR5 | response regulator 5 |
| **Hormone-** **strigolactone** | |  |  |
| MD07G1110600 | AT2G44990.1 | MAX3 | carotenoid cleavage dioxygenase 7 |
| MD15G1057600 | AT2G26170.1 | MAX1 | cytochrome P450, family 711, subfamily A, polypeptide 1 |
| MD08G1084800 | AT3G03990.1 | D14 | alpha/beta-Hydrolases superfamily protein |
| MD17G1266700 | AT2G42620.1 | MAX2 | RNI-like superfamily protein |
| MD06G1211100 | AT3G18550.1 | BRC1 | TCP family transcription factor |
| MD16G1049000 | AT1G68800.1 | BRC2 | TCP domain protein 12 |
| **Axillary meristem** | |  |  |
| MD11G1253500 | AT5G53950.1 | CUC2 | NAC (No Apical Meristem) domain transcriptional regulator superfamily protein |
| MD03G1045700 | AT1G55580.1 | LAS | GRAS family transcription factor |
| MD06G1169000 | AT5G63090.1 | LOB | Lateral organ boundaries (LOB) domain family protein |
| MD15G1302300 | AT3G49690.1 | RAX3 | myb domain protein 84 |
| MD03G1118500 | AT5G60690.1 | REV | Homeobox-leucine zipper family protein / lipid-binding START domain-containing protein |
| MD05G1352500 | AT1G62360.1 | STM | KNOX/ELK homeobox transcription factor |
| **Cell proliferation and growth** | |  |  |
| MD08G1092900 | AT5G67260.1 | CYCD3;1 | CYCLIN D3;1 |
| MD15G1313500 | AT5G65460.1 | KAC2 | kinesin like protein for actin based chloroplast movement 2 |
| MD07G1045800 | AT3G12280.2 | RBR | retinoblastoma-related 1 |
| MD03G1078300 | AT2G29570.1 | PCNA2 | proliferating cell nuclear antigen 2 |
| MD13G1237300 | AT4G25810.1 | XTH23 | xyloglucan endotransglycosylase 6 |
| MD16G1267200 | AT5G57560.1 | TCH4 | Xyloglucan endotransglucosylase/hydrolase family protein |

Supplementary Figure S4. Expression of SL synthesis and signaling (A), CK synthesis and signaling, and auxin transport (B) related genes in root during axillary bud activation. Data are means ± SE (*n* = 3 replicates). Significant differences (***P* < 0.01, **P* < 0.05) are based on paired samples *t*-test.


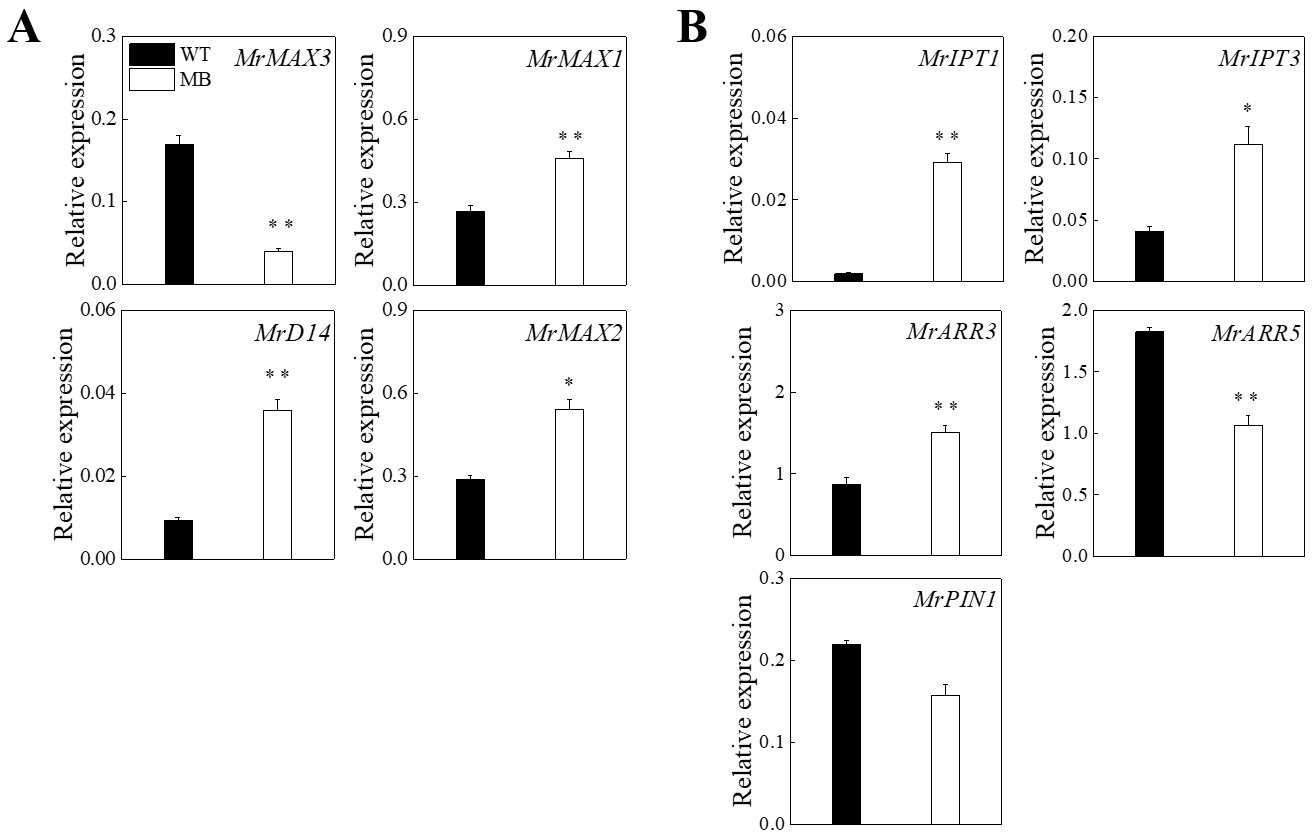


Supplementary Figure S5. Bud length in response to GR24 treatment. (A) Bud in WT, T337, M26, and Nagafu 2. (B) Bud phenotypes of T337. Data represent the mean ± SE (*n* = 10).


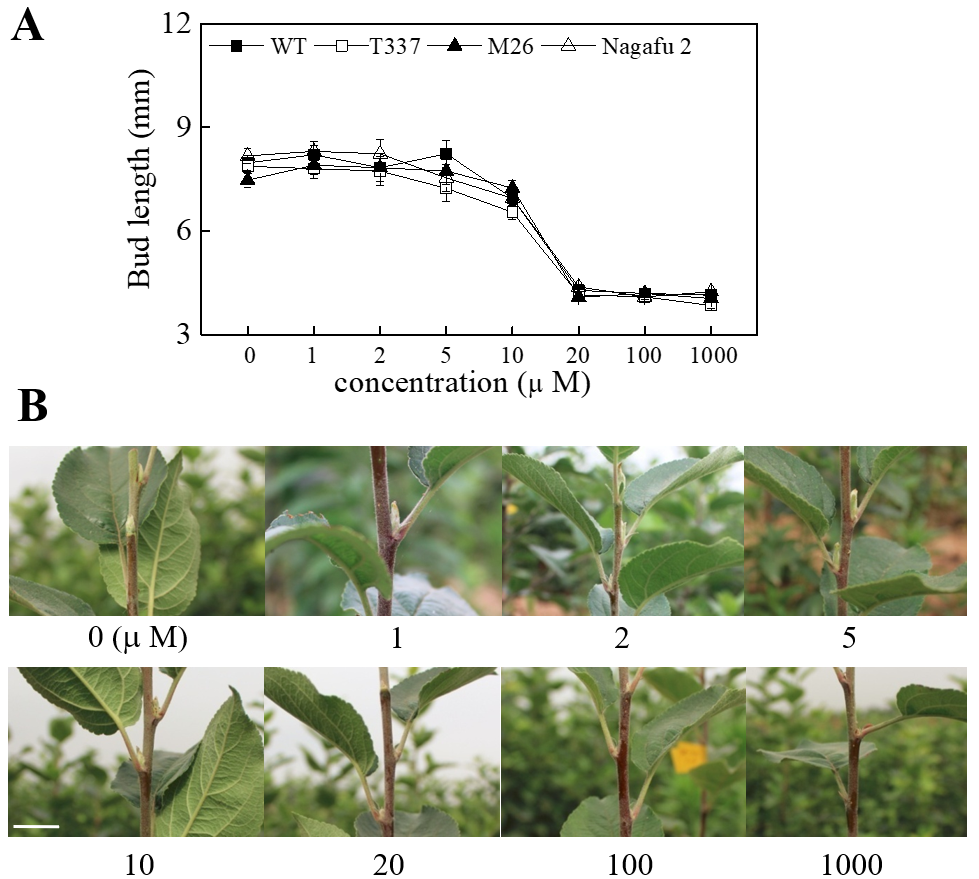


Supplementary Figure S6. Gene expression of the WT axillary buds in response to 6-BA and GA_3_ treatments. Data represent the mean ± SE (*n* = 3 replicates). Control represents no treatment on intact plants. Lowercase letters indicate significant differences by a Student-Newman-Keuls test (*P* < 0.5).


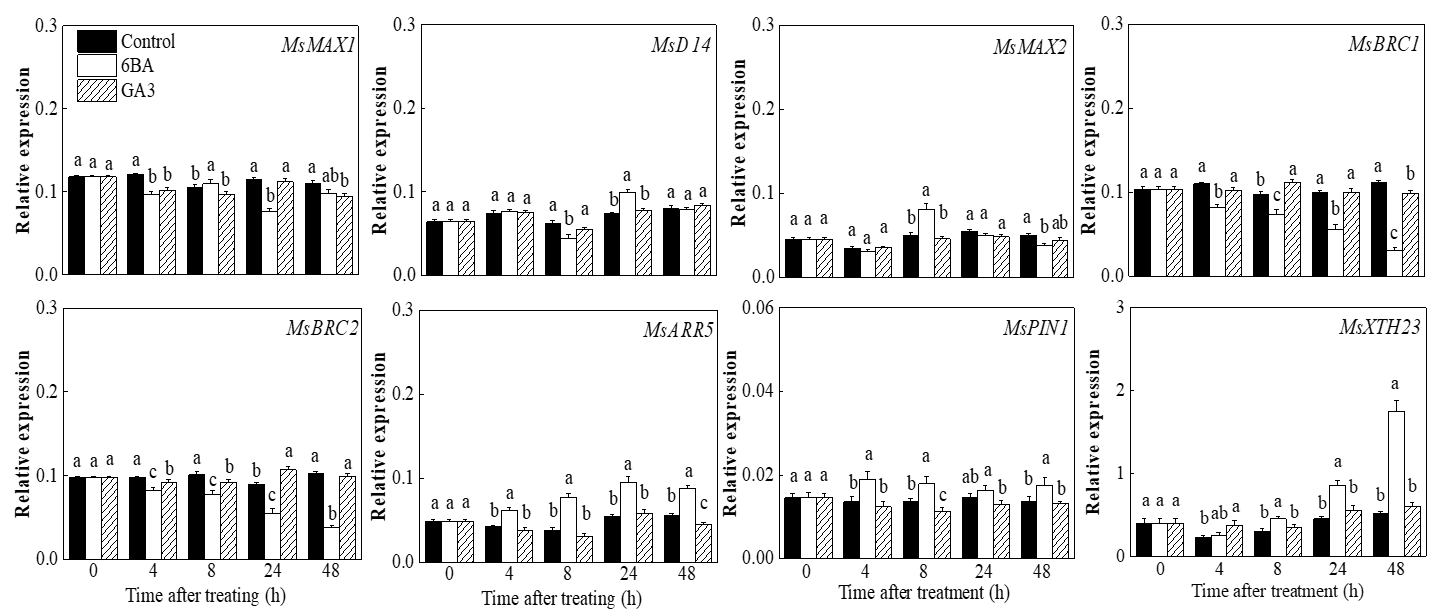

Supplement: Supplementary file 1 [file Table_1.doc]
